# Design and Validation of a Questionnaire on Risk Perception, Coping Behaviors and Preventive Knowledge against COVID-19 among Nursing Students

**DOI:** 10.3390/jpm12040515

**Published:** 2022-03-22

**Authors:** José Rafael González-López, Diego Serrano-Gómez, Verónica Velasco-González, Ana Rosa Alconero-Camarero, Daniel Cuesta-Lozano, Esther García-García, Pilar González-Sanz, Iván Herrera-Peco, Esther Martínez-Miguel, José María Morán-García, José Ignacio Recio-Rodríguez, Carmen Sarabia-Cobo

**Affiliations:** 1 Department of Nursing, Faculty of Nursing, Physiotherapy and Podiatry, Universidad de Sevilla, 41009 Seville, Spain; joserafael@us.es; 2 Faculty of Health Sciences, Universidad de Burgos, 09001 Burgos, Spain; dserrano@ubu.es; 3 Department of Nursing, Faculty of Nursing, Universidad de Valladolid, 47005 Valladolid, Spain; 4 IDIVAL Nursing Research Group, Faculty of Nursing, Universidad de Cantabria, 39008 Santander, Spain; ana.alconero@unican.es (A.R.A.-C.); carmen.sarabia@unican.es (C.S.-C.); 5 Department of Nursing and Physiotherapy, Faculty of Medicine and Health Sciences, Universidad de Alcalá, 28871 Madrid, Spain; daniel.cuesta@uah.es; 6 Department of Nursing and Nutrition, Faculty of Biomedical and Health Sciences, Universidad Europea de Madrid, 28670 Madrid, Spain; esther.garcia@universidadeuropea.es (E.G.-G.); pilar.gonzalez@universidadeuropea.es (P.G.-S.); esther.martinez@universidadeuropea.es (E.M.-M.); 7 Nursery Department, Faculty of Medicine, Universidad Alfonso X el Sabio, 28911 Madrid, Spain; iherrpec@uax.es; 8 Metabolic Bone Diseases Research Group, Nursing and Occupational Therapy College, Universidad de Extremadura, 10003 Cáceres, Spain; jmmorang@unex.es; 9 Faculty of Nursing and Physiotherapy, Universidad de Salamanca, 37007 Salamanca, Spain; reciorodriguezji@gmail.com

**Keywords:** nursing education, nursing students, risk assessment, coping behavior, knowledge, COVID-19, validation study, psychometric

## Abstract

As shown in the previous literature, in view of the future responsibilities of nursing professionals and the consequences for healthcare, it is of great interest to examine their risk perceptions, coping behaviors, and sense of coherency during the COVID-19 pandemic. The purpose of this study is to design and to validate a specific questionnaire that evaluates the factors relating to perceived risk, coping behaviors, and preventive knowledge against COVID-19 infection among nursing students from Spain. This is a psychometric study of a questionnaire’s design and its validation in 1562 nursing students at 16 undergraduate nursing institutions in Spain. An *ad-hoc* survey was designed by a panel of six experts drawing from the literature. After a trial test, the questionnaire was formed with four scales (perception, risk, coping, and knowledge of preventive practices for COVID-19), with a total of 69 items. The final questionnaire was composed of 52 items grouped into four scales, with good psychometric properties to measure risk perception (Cronbach’s alpha 0.735), factors related to perceived risk (Cronbach’s alpha 0.653), coping behaviors (Cronbach’s alpha 0.80), and knowledge of preventive practices against COVID-19 (Cronbach’s alpha 0.77). This questionnaire, specifically designed and validated for nursing students, is the first to address four important areas in the development of preventive measures against COVID-19.

## 1. Introduction

The SARS-CoV-2 pandemic has caused profound upheaval in society, as the routine behavior of the population must to guided towards safer, preventive, and cooperative behaviors [1]. The risks of infection, illness, serious illness, and even death, as well as the risk of becoming a vector of infection (endangering family and community networks) are decisive factors for changes to individual, social, and even professional contact. In these reasonable and safe behaviors, the perception of risk is a key element for the development of reasonable risk management behavior [2].

The perception of risk is explained in different theoretical models. According to the Health Belief Model (HBM), for example, people tend to have a lower perception of risk, as a consequence of what they consider to be illusory optimism [3,4]. Among other factors, perception of vulnerability, susceptibility, self-efficacy, and locus of control are complex constructs that make it difficult for professionals to alter risk behaviors to improve populational health [5,6]. In that sense, risk may be considered a first-level stress factor. The salutogenic model is a model that explains how each person can self-manage stressful situations [7]. In this model, a key concept is Sense of Coherency (SOC), according to which the use of personal, interpersonal, and community resources (Generalized Resistance Resources) available to people and communities develop their cognitive, psychomotor, and emotional strategies to cope with stressful situations better, improving their resilience and their own effectiveness.

In investigating risk perceptions, the coping behaviors and the SOC of future nursing professionals during the COVID-19 pandemic is of great interest, because a previous study [8] warned of nursing responsibility in nurturing professional care and individual and community self-care, which includes awareness raising and the reduction of risk-exposure through strengthening self-management of health. In consequence, from the perspective of health psychology and the HBM, we will design specific strategies to ensure control over self-efficacy and to develop realistic coping mechanisms [9].

To date, no specific scale has been designed to measure risk perception, coping behaviors and preventive knowledge against COVID-19 among young people. Neither is there, to the best of our knowledge, a study of these dimensions among nursing students within one country.

The objective of this study is therefore the design and the validation of a specific questionnaire that evaluates perceived risk, coping behaviors, and preventive knowledge against COVID-19 infection among nursing students within Spain.

## 2. Methods

### 2.1. Design

We performed a psychometric study of a questionnaire’s design and its validation, evaluating risk perception, risk factors, coping behaviors, and preventive knowledge against COVID-19. Data collection took place between October 2020 and January 2021.

The individuals were nursing students across all years of nursing degree courses at 13 Spanish universities. The only inclusion criterion was to be a student of a nursing degree. There were no exclusion criteria. In Spain, the nursing degree is of a four-year duration and the students enter clinical practice at health institutions in their second year. Data collection was anonymous and held no academic consequences for students. No negative consequences ensued from non-participation.

### 2.2. Sampling Size

Intentional sampling selection was used. A sample of 336 individuals for a sample universe of 7.479 students was sufficient to estimate a representative mean population at a confidence level of 95% and with an error margin of 5.

### 2.3. Design of the Scale

Drawing from the literature [10,11,12,13], an *ad-hoc* questionnaire was designed by a panel of six experts for the evaluation of risk perception, factors relating to perceived risk, coping behaviors, and preventive knowledge against COVID-19. The process of construction, piloting, and validation of the instrument was carried out following the recommendations of Cook and Beckman [14]. The expert panel was composed of psychologists, one specializing in health psychology and the other in behavioral change, and two specialist nurses, one trained in family and the other in community nursing. The survey was given the title: Questionnaire on Perception, Risk, Coping, Knowledge, COVID-19 (PRCK-COVID-19).

A group evaluation of the psychometric characteristics (validity, reliability, precision, etc.) necessary for the future questionnaire was conducted in three sessions. The steps described by Dawis were followed [15]. Four specific scales were initially drafted by area: risk perceptions, factors relating to perceived risk, coping behaviors and preventive knowledge against COVID-19, with a total of 71 items and a 1–5-point Likert-type response scale. The questionnaire was initially administered to a sample of 30 nursing students for exploratory purposes. After its first use, the degree of comprehension of each item was evaluated and questions and statements from each item were reviewed. Similar questions or questions evaluating the same area or function were removed and confusing or inappropriate questions were reworded. A total of 69 items were generated in this process for all four scales. The final version underwent a process of qualitative assessment of each item, agreed to within the working group. Each item was differentiated as “good”, “regular”, and “bad”, as a function of its capability to evaluate each specific area in the scale in accordance with the experience of each professional in the group. Following the assessment, the final version was reviewed. Items were repositioned according to the most appropriate dimension, reworded to improve comprehension, and clarifications and examples were in some cases added and in other cases removed. The process ended with the coding of all items and sections with their definitive formulation, achieving the final coded version of four scales with 66 items. Following the design process described above, the final version of the PRCK-COVID-19 questionnaire presented the following four scales:

- Scale of Perceived Risk. This scale refers to the global perception of the COVID-19 infection risk. The person has to select the risk level of infection from 3 phrases that are presented (0—none; 10—maximum risk). The maximum score was 30 points, indicating higher perceived COVID-19 infection risks through higher scores;
- Scale of Factors Relating to Perceived Risk. Initially formed of 20 items, this scale establishes the risk factors of infection according to the degree of agreement with the items that are presented. It is more specific than the earlier scale, employing a Likert-type scale (1—totally disagree; 5—totally agree);
- Scale of Coping Behaviors against infection. Initially formed of 24 items, each question inquires into individual coping behaviors against the risk of COVID-19 infection. A Likert-type scale (1—totally disagree; 5—totally agree) was used in which the respondent expresses a degree of agreement or disagreement with the items that have been presented. Eight of the items were inverse.
- Scale of Preventive Knowledge against COVID-19. Initially formed of 19 items, this scale reflects the degree of agreement with the items that refer to preventive measures against infection (1—totally disagree; 5—totally agree).

### 2.4. Procedure

All the variables were integrated in an *ad-hoc* questionnaire design administered through an online platform. The online “Google Forms” tool was used for the survey design. An introductory letter was sent to all participating centers requesting their collaboration and explaining the purpose of the study with a description of the questionnaire (including its estimated completion time and number of items), as well as information on the project research team and the link to access it. The questionnaires were sent through the email lists with the previous agreement of the educational center and the responses were controlled through an IP response identification protocol to avoid multiple responses.

### 2.5. Statistical Analysis

IBM SPSS Statistics 26 and AMOS were employed as the statistical packages. The construct validity of this question was calculated by means of factor analysis through principal component extraction and Kaiser Varimax rotation, for each of the four scales. Each variable was included as a single factor, attending to factor loading, setting values of 0.30 as a minimum saturation criterion. Varimax rotation was considered as the most appropriate, given that it is expected to identify the maximum number of factors that form each scale. The Kaiser–Meyer–Olkin (KMO) estimators of sampling adequacy (range between 0–1) and Bartlett’s test for homogeneity of variances significance were calculated (if their value is close to a single unit and they are significative to 0 > 0.05, the analysis with reduction of variables is adequate). The reliability of the questionnaire was calculated through the analysis of internal consistency, for which purpose Cronbach’s Alpha was used, which should be interpreted as an indicator of the internal consistency and therefore reliability of the items, as it reflects the variance between the individual items and their total scores on each scale.

Exploratory Factor Analysis (EFA) was used to analyze the correlations, reliability and construct validity, thereby testing the aprioristic model. The items validated through factor saturation were then selected for the predictive model.

### 2.6. Ethical Considerations

The researchers reported no conflict of interests. Approval was granted from the Project Ethics Committee of the University of Cantabria (EC Projects, 13/2020). Data processing methods guaranteed the confidentiality of the data and study-related information, in fulfilment of current Spanish legislation according to the General Data Protection Regulation (Reglamento General de Protección de Datos) (RGPD), approved on 25 May 2018, in substitution of Organic Law 15/1999, of 13 December, on the protection of physical persons with respect to the treatment of personal data. The principal researcher is the person responsible for guaranteeing that the data cannot be used for any other purpose. Before beginning the questionnaire, following the letter of presentation of the study, the respondent had to tick a specific box, thereby consenting to participation in the study, which may be corroborated from the completed questionnaires.

## 3. Results

A total of 1562 people responded to the questionnaire. In all, 87.5% were women (*n* = 1366), with an average age of 21.5 years (SD 5.7). Distribution by year was as follows: first year (*n* = 432), 27.7%; second year (*n* = 413), 26.4%; third year (*n* = 346), 22.2%; and fourth year (*n* = 355), 22.7%.

All the experts considered that the instrument and both its qualitative and its quantitative contributions to content validity were very appropriate. In particular, some minimum values of Aiken’s V coefficient for the four scales were obtained for both content and form: 0.81 and 0.90, respectively, which are very acceptable values.

Each scale of the questionnaire was independently analyzed, applying the same analysis procedure to them all.

The Scale of Perceived Risk was formed by three items. The factor analysis identified a single-factor structure (Kaiser–Meyer–Olkin sampling adequacy measurement of 0.65, Bartlett’s sphericity test *p* < 0.001, correlations matrix determinant <0.001), which explained 65.3% of the variance. Each item was included in the single factor in accordance with its factoring load, with saturation values higher than 0.70. The VARIMAX factor rotation solutions formed a well-defined structure with no overlap. The questionnaire results yielded a Cronbach’s alpha value of 0.735. This value that was not improved after deleting some of the items; therefore, they were all maintained. The item-total correlation values fluctuated between 0.52 and 0.73. All the questions were answered by 99% of the students.

The Scale of Factors Relating to Perceived Risk was formed initially of 20 items. The factor analysis identified a two-factor structure (Kaiser–Meyer–Olkin sampling adequacy measurement of 0.78, Bartlett’s sphericity test *p* < 0.001, matrix correlation determinant <0.001), which explained 51.2% of the variance. Each item was included in a single factor with respect to its factoring load, with saturation values greater than 0.40. It was decided to exclude four items, so as not to saturate any of the factors. The VARIMAX factor rotation solutions constituted a well-defined structure with no overlaps. After the exclusion of four items, the questionnaire results yielded a Cronbach’s alpha of 0.653. The item-total correlation values fluctuated between 0.52 and 0.73. All the questions were answered by 99% of the students. The two factors that were identified related to how the person perceived the risk factors: either as their own and therefore dependent on the way they were acting (intrinsic factor, FR1) or as external risk factors and dependent upon the environment (extrinsic factor, FR2). Finally, this scale was composed of 16 items. The first factor, the intrinsic factor, contained seven items and explained 36% of the variance. The second factor, the extrinsic factor, included nine questions and explained 15.2% of the variance. The final scale was therefore left with 16 items and two types of risk factors (intrinsic and extrinsic).

The Scale of Coping Behaviors against infection was initially formed of 24 items. The factor analysis identified a three-factor structure (Kaiser–Meyer–Olkin sampling adequacy of 0.87, Bartlett’s sphericity test *p* < 0.001, correlations matrix determinant <0.001), which explained 47.7% of the variance. Each item was included in the single factor in view of its factoring load, with saturation values higher than 0.40. It was decided to exclude five items, so that none of the factors were saturated. The VARIMAX factor rotation solutions yielded a well-defined structure with no overlap. After the exclusion of five items, a Cronbach’s alpha value of 0.80 was obtained for the questionnaire. The item-total correlation values fluctuated between 0.52 and 0.73. All the questions were answered by 99% of students. Once identified, the three factors related to three coping behaviors against COVID-19: reality-centered, avoidance, and search for support. The first factor, referred to as reality-centered, contained seven items and explained 18.4% of the variance. The second factor, avoidance, was established through seven questions and explained 17.3% of the variance. The third factor, search for support, was defined with five items and explained 12% of the variance. The final scale was therefore composed of 19 items and three coping behaviors against COVID-19: EA1—reality-centered (seven items), EA2—avoidance (seven items), and EA3—search for support (five items). The higher the score, the greater the weight attached to one risk factor than another. From among the coping behaviors that coincided with the literature, reality-centered (greater self-efficacy) and search for support [16] were both preferable.

The Scale of Preventive Knowledge against COVID-19 was initially formed of 19 items. The factor analysis identified a single-factor structure (Kaiser–Meyer–Olkin sampling adequacy measurement of 0.71, Bartlett’s sphericity test *p* < 0.001, matrix correlation determinant *p <* 0.001), which explained 54% of the variance. Each item was included in the single-factor in view of its factoring load, with saturation values greater than 0.40. All the items that were saturated in this single factor, except for Item 5, were removed. The VARIMAX factor rotation solutions formed a well-defined structure with no overlap. The questionnaire results yielded a Cronbach’s alpha value of 0.77. The item-total correlation values fluctuated between 0.52 and 0.83. All the questions were answered by 99% of the students. The single factor that was identified was termed Knowledge. The final scale was composed of 14 items.

In Table 1, all the factorial weights of each scale are shown, grouped under their corresponding factors.

The questionnaire was therefore formed of 52 final items grouped into four scales, with good psychometric properties, in order to measure perceived risk, factors relating to perceived risk, knowledge of preventive behavior, and coping behaviors against COVID-19.

## 4. Discussion

The design and validation of the specific instrument described in this paper was to evaluate perceived risk, factors relating to perceived risk, coping behaviors, and preventive knowledge against COVID-19 among nursing students. We can say that the objective behind the design of the PRCK-COVID-19 questionnaire with good psychometric properties has been achieved.

Factorial analysis through item discrimination was applied to confirm the construct validity of the instrument and to verify its internal consistency with Cronbach’s alpha. Four valid and reliable scales were obtained for nursing students: the Scale of Perceived Risk (α = 0.735), the Scale of Factors Relating to Perceived Risk (α = 0.781) with two risk factors (intrinsic and extrinsic), the Scale of Coping Behaviors (α = 0.889) with three strategies (reality-centered, avoidance, and search for support) and the Scale of Preventive Knowledge (α = 0.81).

If we examine the scales that presented different factors, we will see that these are congruent with the theoretical framework and with other similar scales.

The scale Factors Relating to Perceived Risk presents two types of risk factors: extrinsic and intrinsic. Risk perception means we can assess why people will or will not take steps to protect themselves against external threats [17]. Faced with this fact, a person can perceive the risks as either intrinsic (“I can control my behavior to avoid risk factors”) or as extrinsic (factors beyond individual control and therefore inevitable). The questionnaire included items that captured the seriousness with which the COVID-19 pandemic is perceived, the perceived probability of contracting the virus, the perceived probability of family members and friends contracting the virus, and their actual level of concern over the virus. Our identification of two factors is aligned with other studies similar to our own [18,19], in which these two types of perception can be distinguished with the scales. Similar results were found in studies with Belgian [20] and Saudi [21] nursing students. We adopted a theory-based approach for the study of risk perception employed by Van der Linden [5], a model in which the inclusion of groups of variables is recommended, associated with the cognitive tradition (for example, risk-related knowledge and comprehension among people), emotional and experiential traditions (for example, personal experience), the socio-cultural paradigm (for example, social expansion of the risk, cultural theory, confidence, and values) and relevant individual differences (for example, gender, education, and ideology). In a recent study [22], investigators assessed perceptions of the risk of COVID-19 infection within ten European countries, employing a specific risk perception scale with a similar number of factors to our own.

As regards the Scale of Coping Behaviors against COVID-19, the factorial analysis pointed to three strategies: reality-centered, avoidance, and search for support. The desirable coping behaviors are reality-centered, which refers to how the person in a practical and realistic manner decides to take preventive measures, and search for support, which is centered on the search for expert opinion and social support for decision-making [23]. The least desirable strategy is avoidance, which refers to a negation of reality and an avoidance of the problem, either refusing to think of it or refusing to take preventive decisions [24]. These results are in agreement with Lazarus and Folkman’s transactional theory of stress and coping [25], which identifies two strategies: problem-focused and emotion-focused. The first is a problem-focused strategy that coincides with our own; these are behaviors directed towards the definition of the problem, the search for alternative solutions, the consideration of cost-benefit-based alternatives, and their selection and application. In turn, the emotion-focused strategy, according to these authors [25], has the function of emotional regulation that includes efforts to reduce unease and to manage emotional states caused by stressful events. In general terms, these objectives may be achieved by avoiding the stressful situation, cognitively revaluating the disturbing event, or selectively looking at positive aspects of oneself or the environment. Likewise, according to the theoretical model of Lazarus and Folkman [25], the aforementioned strategy includes various categories such as social emotional support, negation, and liberation. Our three-factor scale, therefore, fits with the most extensive model of these coping behaviors [26]. As far as we know, previously validated scales on coping behaviors have been employed, although they are generic and are not specific to the COVID-19 situation, unlike our study [27,28].

The Scale of Preventive Knowledge against COVID-19 also presented good reliability and internal consistency. Its items and objectives were similar to other studies, in which other knowledge-specific and preventive behavior scales against the virus have also been presented [21,29].

The second part of this study, which will be published in another article, presents the most significant descriptive results, as well as the development of a predictive model to establish relationships between student characteristics (age, whether the student has suffered from the disease, etc.) and the results on the scales. This instrument has practical applications not only related to COVID-19, but also to other contagious diseases or other health risk behaviors. Its use can be immediate in other places and countries, after a process of cultural adaptation and translation.

This study has some limitations. On the one hand, the results of the study may not be generalized to other countries, even though the large sample size of the present study is representative of the Spanish university population. On the other hand, this study was conducted in the very early stages of the pandemic in Spain, at a time where scientific knowledge on the virus was limited.

## 5. Conclusions

In so far as we are aware, the PRCK-COVID-19 questionnaire specifically designed for nursing students is the first in which four important areas are considered that cover the development of preventive measures against COVID-19: perceived risk, factors relating to perceived risk, coping behaviors, and preventive knowledge. We have applied both (quantitative and qualitative) face validity and content validity methods, construct validity using exploratory factor analysis, and scale reliability using two methods of internal consistency and stability. The psychometric properties were good and their use will be beneficial for the detection of students with low-risk perceptions, coping behaviors of avoidance, and limited preventive knowledge against the virus. All this means that educational measures and preventive management of the situation may be developed within a salutogenic model, especially when the students perform their clinical practice.

## Figures and Tables

**Table 1:** Factorial weights of each item by scale and factor (PCA rotation).

**Scale of Perceived Risk**			
**Items**	**Perceived Risk Factor**		
How likely do you think it is that you could be infected with COVID-19 (Coronavirus SARS-CoV-2) in the near future?	0.816		
What likelihood is there that people from your family and friends may be infected with COVID-19 in the near future?	0.859		
How likely do you think infection by COVID-19 will be in general?	0.745		
**Scale of Factors relating to Perceived Risk**			
**Items**	**Intrinsic Factor**	**Extrinsic** **Factor**	
Being infected with COVID-19 concerns me.	**0.843**	0.155	
Everything related to COVID-19 frightens me.	**0.71**	0.282	
I couldn’t care less about infection with COVID-19.	**0.696**	0.014	
I am very concerned that somebody close to me may become infected.	**0.605**	0.011	
I can easily reduce the dangers of infection.	**0.805**	−0.076	
How serious the consequences of COVID-19 will be for me all depends on myself.	**0.684**	−0.17	
COVID-19 is not going to affect me.	**0.401**	−0.116	
COVID-19 will become increasingly dangerous over time.	0.151	**0.697**	
COVID-19 will continue to affect future generations.	−0.016	**0.622**	
COVID-19 will cause many more deaths at the same time.	0.101	**0.582**	
If I am infected by COVID-19, I think I could die.	0.326	**0.505**	
Those who have died because of COVID-19 were already ill beforehand.	0.039	**0.679**	
The experts know how to manage COVID-19.	0.08	**0.646**	
The communications media have exaggerated the consequences of COVID-19.	−0.031	**0.789**	
The risks associated with COVID-19 have different effects according to the environment in which you are living (poverty, among others).	0.215	**0.589**	
COVID-19 is something completely new for us all.	0.113	**0.566**	
**Scale of Coping Behaviors**			
**Items**	**Reality-** **Centered**	**Avoidance**	**Search for** **Support**
I concentrate on what to do next.	**0.724**	0.016	0.131
I try not to do anything imprudent.	**0.723**	−0.110	0.034
I know what to do and try to follow it strictly.	**0.706**	−0.062	0.253
I change things in my life to be able to cope with it better.	**0.586**	−0.012	0.337
I have repeatedly thought about it and I try to understand it all in as far as may be possible.	**0.515**	0.003	0.358
I listen to experts and follow their advice.	**0.509**	−0.101	0.222
I concentrate on what to do afterwards when it is all over.	**0.390**	0.264	0.105
I take refuge in dreams and I imagine moments when everything was better than today.	0.008	**0.741**	0.028
I try to leave everything behind and I want to relax or to go on holiday.	−0.026	**0.699**	−0.028
I try to feel better eating, drinking, smoking or taking medication due to anxiety and nervousness.	−0.072	**0.586**	0.045
I’m hoping for a miracle that will solve everything.	0.020	**0.578**	−0.068
I refuse to think that it is happening.	−0.184	**0.549**	−0.021
I wish I could change my concerns and feelings.	0.069	**0.518**	0.119
I imagine how everything might turn out.	0.014	**0.407**	0.162
I ask for advice from highly respected people and I keep to it.	0.207	0.025	**0.773**
I talk to someone who is knowledgeable of the subject.	0.176	−0.070	**0.768**
I talk with others to learn more about their circumstances.	0.315	−0.014	**0.618**
I am doing something completely new that I had never done under other circumstances.	0.127	0.182	**0.510**
I have been thinking of what is usually done in relation to other viral infections, such as the ‘flu’.	0.213	0.034	**0.395**
**Scale of Preventive Knowledge**			
**Items**	**Knowledge** **Factor**		
It is important to prepare yourself and to buy food in large quantities (flour, sugar, pasta, rice, tinned food) due to possible COVID-19 (Coronavirus SARS-CoV-2) lockdowns.	0.801		
Buy large amounts of toilet paper and other items for personal cleanliness and other hygiene articles, thinking of lockdowns within the near future.	0.781		
I trust in hospitals, emergency services and professionals who are helping me to protect myself.	0.759		
I trust in the authorities and medical experts and I follow their indications.	0.74		
Use gloves every time you go out on the street.	0.609		
Avoid public places/events.	0.543		
Maintain a minimum safety distance within public spaces of at least one and a half meters at least.	0.523		
Avoid visits from other people to the house except for essentials.	0.505		
At all times, hydroalcoholic lotions must substitute water and soap.	0.5		
Wash or disinfect the hands more frequently than was usual.	0.454		
Avoid public transport (metro, bus, train).	0.446		
I have bought large amounts of disinfectant/hand soap.	0.441		
Avoid contact with risk groups (older people, people with previous illnesses).	0.434		
I use a face mask whenever I go out on the street.	0.434		

Extraction method: PCA analysis. Rotation method: Kaiser varimax rotation with Kaiser normalization.

## Data Availability

The data presented in this study are available on request from the corresponding author.

## References

[1] Xu H., Mendez M.J.G., Guo L., Chen Q., Zheng L., Chen P., Cao X., Liu S., Sun X., Zhang S. (2020). Knowledge, Awareness, and Attitudes Relating to the COVID-19 Pandemic Among Different Populations in Central China: Cross-Sectional Survey. J. Med. Internet Res..

[2] Zhong B.L., Luo W., Li H.M., Zhang Q.Q., Liu X.G., Li W.T., Li Y. (2020). Knowledge, attitudes, and practices towards COVID-19 among Chinese residents during the rapid rise period of the COVID-19 outbreak: A quick online cross-sectional survey. Int. J. Biol. Sci..

[3] Anderson R.M., Heesterbeek H., Klinkenberg D., Hollingsworth T.D. (2020). How will country-based mitigation measures influence the course of the COVID-19 epidemic?. Lancet.

[4] Del Río C., Malani P.N. (2020). COVID-19—New insights on a rapidly changing epidemic. JAMA.

[5] Van der Linden S., Nisbet M.C., Schafer M., Markowitz E., Ho S., O’Neill S., Thaker J. (2017). Determinants and Measurement of Climate Change Risk Perception, Worry, and Concern. The Oxford Encyclopaedia of Climate Change Communication.

[6] Kebede Y., Yitayih Y., Birhanu Z., Mekonen S., Ambelu A. (2020). Knowledge, perceptions and preventive practices towards COVID-19 early in the outbreak among Jimma university medical center visitors, Southwest Ethiopia. PLoS ONE.

[7] Mittelmark M.B., Sagy S., Eriksson M., Bauer G.F., Pelikan J.M., Lindström B., Arild Espnes G. (2017). The Handbook of Salutogenesis.

[8] Rodríguez-Besteiro S., Tornero-Aguilera J.F., Fernández-Lucas J., Clemente-Suárez V.J. (2021). Gender Differences in the COVID-19 Pandemic Risk Perception, Psychology and Behaviors of Spanish University Students. Int. J. Environ. Res. Public Health.

[9] Hargreaves J., Davey C. (2020). Three lessons for the COVID-19 response from pandemic HIV. Lancet HIV.

[10] Brug J., Aro A.R., Oenema A., de Zwart O., Richardus J.H., Bishop G.D. (2004). SARS risk perception, knowledge, precautions, and information sources, the Netherlands. Emerg. Infect. Dis..

[11] De Zwart O., Veldhuijzen I.K., Elam G., Aro A.R., Abraham T., Bishop G.D., Voeten H.A., Richardus J.H., Brug J. (2009). Perceived threat, risk perception, and efficacy beliefs related to SARS and other (emerging) infectious diseases: Results of an international survey. Int. J. Behav. Med..

[12] Gerhold L. (2020). COVID-19: Risk Perception and Coping Strategies (Online). https://psyarxiv.com/xmpk4.

[13] Kim J.S., Choi J.S. (2016). Middle East respiratory syndrome–related knowledge, preventive behaviours and risk perception among nursing students during outbreak. J. Clin. Nurs..

[14] Cook D.A., Beckman T.J. (2006). Current concepts in validity and reliability for psychometric instruments: Theory and application. Am. J. Med..

[15] Dawis R.V., Tinsley H.E.A., Brown S.D. (2000). Scale construction and psychometric considerations. Handbook of Applied Multivariate Statistics and Mathematical Modeling.

[16] Greenaway K.H., Louis W.R., Parker S.L., Kalokerinos E.K., Smith J.R., Terry D.J., Boyle G.J., Saklofske D.H., Matthews G. (2015). Measures of coping for psychological well-being. Measures of Personality and Social Psychological Constructs.

[17] Rana I.A., Bhatti S.S., Aslam A.B., Jamshed A., Ahmad J., Shah A.A. (2021). COVID-19 risk perception and coping mechanisms: Does gender make a difference?. Int. J. Disaster Risk Reduct..

[18] Cori L., Bianchi F., Cadum E., Anthonj C. (2020). Risk perception and COVID-19. Int. J. Environ. Res. Public Health.

[19] De Bruin W.B., Bennett D. (2020). Relationships between initial COVID-19 risk perceptions and protective health behaviors: A national survey. Am. J. Prev. Med..

[20] Ulenaers D., Grosemans J., Schrooten W., Bergs J. (2021). Clinical placement experience of nursing students during the COVID-19 pandemic: A cross-sectional study. Nurse Educ. Today.

[21] Albaqawi H.M., Alquwez N., Balay-odao E., Bajet J.B., Alabdulaziz H., Alsolami F., Tumala R.B., Alsharari A.F., Tork H.M.M., Felemban E.M. (2020). Nursing Students’ Perceptions, Knowledge, and Preventive Behaviors Toward COVID-19: A Multi-University Study. Front. Public Health.

[22] Dryhurst S., Schneider C.R., Kerr J., Freeman A.L., Recchia G., Van der Bles A.M., Spiegelhalter D., Van Der Linden S. (2020). Risk perceptions of COVID-19 around the world. J. Risk Res..

[23] Garbóczy S., Szemán-Nagy A., Ahmad M.S., Harsányi S., Ocsenás D., Rekenyi V., Al-Tammemi A.B., Kolozsvári L.R. (2021). Health anxiety, perceived stress, and coping styles in the shadow of the COVID-19. BMC Psychol..

[24] Yu H., Li M., Li Z., Xiang W., Yuan Y., Liu Y., Li Z., Xiong Z. (2020). Coping style, social support and psychological distress in the general Chinese population in the early stages of the COVID-19 epidemic. BMC Psychiatry.

[25] Lazarus R.S., Folkman S. (1984). Stress, Appraisal and Coping.

[26] Biggs A., Brough P., Drummond S. (2017). Lazarus and Folkman’s psychological stress and coping theory. Handb. Stress Health A Guide Res. Pract..

[27] Huang L., Lei W., Xu F., Liu H., Yu L. (2020). Emotional responses and coping strategies in nurses and nursing students during COVID-19 outbreak: A comparative study. PLoS ONE.

[28] Rogowska A.M., Kuśnierz C., Bokszczanin A. (2020). Examining anxiety, life satisfaction, general health, stress and coping styles during COVID-19 pandemic in Polish sample of university students. Psychol. Res. Behav. Manag..

[29] Soltan E.M., El-Zoghby S.M., Salama H.M. (2020). Knowledge, risk perception, and preventive behaviors related to COVID-19 pandemic among undergraduate medical students in Egypt. SN Compr. Clin. Med..

